# Matrons in Council: III. From an Old Shoe

**Published:** 1917-08-18

**Authors:** 


### THE MATRONS' AND SISTERS' DEPARTMENT.

### MATRONS IN COUNCIL.

### III. From an Old Shoe.

Do not the Sparks fly a little overmuch? Let us see how the Nails can be driven Home.

That it would be a good thing that all Branches of the Nursing Profession (not excluding the Navy and Army) be associated in efforts towards a thorough Reorganisation of the Profession is a great point to drive Home, but it is rather a large order to ask the Matrons to force this, seeing the Material is not yet ready.

The Matter lies deeper than any "Levelling" or "Pooling," or the putting forth of any additional comfort or sympathy. The Trained Nurse does not ask for Mothering. It is a small affair.

A Radical Change is needed, but an upheaval must come first.

Whilst every Hospital frames its own Laws (and systems are many), and the Governors are striving to make the Income match Expenditure, the present Anomalies will persist.

However High the Ideal, Stern Necessity rules. To bring us into Line with America and the Dominions more generous ways of providing for Improvements must be forthcoming—a graduated Scale of Payments from Patients, for instance.

It is wonderful how well, on the whole, Hospitals work even under past and present Hardships, and what Noble Nurses are built up therein. The War has brought this out. Still we can do better.

Who will Roll away the Stone of Custom? The too long Hours of Ward Duty, the often inadequate Teaching and Pay? The Abuses and Oppressions of Juniors at the Hands of Senior Nurses?

A Voice cries aloud for Revision, not only for Hospital Training Schools, but for Workhouse Infirmaries, Fever Hospitals, Asylums of every kind.

Much ground must be stirred before any real alteration can be hoped for.

The Roots under "The Management" are sometimes Old, Tough, and Deep.

The complacent acquiescence in things as they are will have to be given up.

A more comprehensive view of the Needs of the Nurse and what Doctors and the Public ask of her must take the place of believing "All's Well" when the three years' Certificate is signed, accompanied often by too flattering and flowery Testimonials.

Surely, too, the Governing Body should aim at having its Matron one of—if not *the* best Nurse in the Building—and her experience of practical Work amongst the Sick should neither be "Short" nor Limited. The term "Sister-Matron" is one to be proud of.

No Tribunal can judge Character. The Simplest can possess it—the Wisest be without.

The Loving, Conscientious Soul—more particularly if associated with the Clear Brain, will as ever sweeten even Registration, and not even a College which gains the hoped-for "Royal Educational Establishment" can do more.

Let Matrons, Sisters, Nurses co-operate as far as is possible towards Unity of Ideals—Showing a good front.

Education may help this, although it does not always develop Quality of Character, for that is curiously set apart, belonging to the more Spiritual side of our Nature.

Probably there is some divergence from Florence Nightingale Ideas, but she might well be proud of a multitude of nurses, sent out by our Matrons, well able to cope with these last three strenuous years. There must be a good foundation for Building on presently. Which of us does not know many we could name who could scarcely be improved upon?

Although no Decoration is worn—or paper written on Ideals—let us be grateful for these Nurses—some of them "Matrons" before being "called up" for the work all Love best, and let us wish the Scheme of Reconstruction and Uplifting the best of consultations, with Members from all Branches.

Nursing is as old as Motherhood, but the Profession of it is yet in its Infancy.

The Mother-Spirit of Tenderness for the weak should inspire the future Leaders, so that the Sick are the first consideration.

Perhaps some Nurses are inclined to think their "Rights" (?) are of most importance.

The Spirit of Reform is abroad, the coming of American and Dominion Nurses enlargening—but Time is needed for careful loosening of old foundations.

### Miss L. V. Haughton.

Everybody throughout the nursing world, and a multitude of people, will hear with pleasure that Miss L. V. Haughton's health is steadily improving. This means much, and it is more that *Guy's Hospital Gazette* is able to affirm that she is thoroughly enjoying the beautiful country and fine views of the Surrey hills, where she has been staying for some weeks. Miss Haughton will be cheered and encouraged by a letter which she received from Mrs. Cosmo Bonsor, the President of Guy's Hospital Past and Present Nurses' League, enclosing a cheque for her acceptance, as a declaration of the affection and esteem with which she is regarded, of gratitude for her services to the hospital and the nursing profession, and an expression of deep regret at her serious and untimely illness. The personal

☞ Articles I. and II. appeared August 4 and 11.

character and value of the gift was enhanced by a simply bound manuscript book containing a list of more than 700 names, which had been written as a labour of love in the matron's office. As a frontispiece, the first page contained an intimation that the signatures included present sisters and hospital and institution nurses, the administrative staff, Past and Present League members, friends interested in the Nurses' League, members of the staff, and others who have met Miss Haughton while matron of the hospital, and enjoyed the privilege of her friendship, interest, or advice.

Few, if any, of all the workers for the war have unsparingly rendered the amount of continuous, exacting labour which Miss Haughton devoted to her work as matron of Guy's, in fulfilment of the many claims which devolved upon her in this important and responsible post. Miss Haughton has written a strikingly appreciative letter of thanks, in which she emphasises the honour in which she held her position as matron, and the solace that she has derived, during her illness and severance from the work she loved, from the devotion and remembrance of her friends. We endorse the wish and hope of everyone who knows the facts, and Miss Haughton's career, that she may recover complete health and strength. Long may Miss Haughton be spared to devote her mind and energies to advancing the best interests of the profession which she loves. To the development of the nursing profession she bent her mind and energies, with such devotion, as to overtax her strength. Miss Haughton has proved, nevertheless, that the will to do great deeds makes great deeds done.

